# Detailed statistical analysis plan for a randomised controlled trial of the effects of a modified muscle sparing posterior technique (SPAIRE) in hip hemiarthroplasty for displaced intracapsular fractures on post-operative function compared to a standard lateral approach: HemiSPAIRE

**DOI:** 10.1186/s13063-022-06790-z

**Published:** 2022-11-05

**Authors:** Susan Ball, Amy McAndrew, Alex Aylward, Emma Cockcroft, Elizabeth Gordon, Alison Kerridge, Sarah Morgan-Trimmer, Roy Powell, Anna Price, Shelley Rhodes, Andrew J. Timperley, Jayden van Horik, Robert Wickins, John Charity

**Affiliations:** 1 Applied Research Collaboration South West Peninsula (PenARC), National Institute for Health and Care Research (NIHR), University of Exeter, Exeter, UK; 2 Exeter Clinical Trials Unit, University of Exeter Medical School, Exeter, UK; 3 Applied Research Collaboration South West Peninsula (PenARC) Patient Engagement Group, National Institute for Health and Care Research (NIHR), University of Exeter, Exeter, UK; 4 Primary Care Research Group, University of Exeter Medical School, Exeter, UK; 5 Research and Development Department, Royal Devon University Healthcare NHS Foundation Trust, Exeter, UK; 6 Department of Health and Community Sciences, University of Exeter Medical School, Exeter, UK; 7 Research Design Service – South West, Royal Devon University Healthcare NHS Foundation Trust, Exeter, UK; 8 Exeter Hip Unit, Royal Devon University Healthcare NHS Foundation Trust, Princess Elizabeth Orthopaedic Centre, Exeter, UK; 9 Physiotherapy, Royal Devon University Healthcare NHS Foundation Trust, Exeter, UK

**Keywords:** Statistical analysis plan, Hip hemiarthroplasty, SPAIRE, Randomised controlled trial, Survivor average causal effect

## Abstract

**Background:**

The HemiSPAIRE trial is being conducted to determine whether a modified muscle sparing technique (SPAIRE-“Save Piriformis and Internus, Repairing Externus”) in hip hemiarthroplasty brings clinical benefits compared to the standard lateral technique in adults aged 60 years or older, with a displaced intracapsular hip fracture. This article describes the detailed statistical analysis plan for the trial.

**Methods and design:**

HemiSPAIRE is a definitive, pragmatic, superiority, multicentre, randomised controlled trial (with internal pilot) with two parallel groups. Participants, ward staff and all research staff involved in post-operative assessments are blinded to allocation. This article describes in detail (1) the primary and secondary outcomes; (2) the statistical analysis principles, including a survivor average causal effect (SACE) method chosen specifically to address the issue of potential bias from differential survival between trial arms, which was seen from data review by the Trial Steering Committee, the participants that will be included in each analysis, the covariates that will be included in each analysis, and how the results will be presented; (3) planned main analysis of the primary outcome; (4) planned analyses of the secondary outcomes; and (5) planned additional analyses of the primary and secondary outcomes.

**Trial registration:**

ClinicalTrials.gov NCT04095611. Registered on 19 September 2019.

## Background

The National Institute for Health and Care Excellence (NICE) recommends hemiarthroplasty as the procedure of choice for the treatment of displaced intracapsular fractures when patients are not eligible for total hip replacement and recommends that hemiarthroplasties are carried out using a lateral approach rather than a conventional posterior approach [1]. Some studies report dislocation in up to 10% of patients using the standard posterior approach and such complications can lead to catastrophic consequences [2]. To address the issue of instability leading to dislocation, modifications in the surgical approaches have been attempted using minimally invasive and muscle-sparing techniques [2]. In 2016, the Hip Unit at the Royal Devon and Exeter NHS Foundation Trust developed a modified technique using a posterior approach, named “SPAIRE”; “Save Piriformis and Internus, Repairing Externus”. The combination of this muscle-sparing approach with enhanced capsule repair aims to provide sufficient stability to enable patients to mobilise full-weight bearing, without the specific restrictions currently included in routine post-operative posterior approach protocols.

The study protocol for the HemiSPAIRE randomised controlled trial was published in 2021 and included a brief overview of the statistical analyses [3]. The International Conference on Harmonisation (ICH) guidelines state that primary statistical analyses should be pre-specified, to prevent data-driven choice of analyses and selective reporting of outcomes [4]. This article presents the detailed statistical analysis plan, which was finalised in August 2022, prior to the final participant follow-up, with the analyses following the Consolidated Standards of Reporting Trials (CONSORT) [5].

## Methods and design

### Brief study overview

The HemiSPAIRE trial is a definitive, pragmatic, superiority, randomised controlled trial (with internal pilot) with two parallel groups and blinded assessment. The study population is adults aged 60 years or older attending one of six hospital sites in the South West of England, with a displaced intracapsular fracture requiring hip hemiarthroplasty. All patients requiring hemiarthroplasty for a displaced intracapsular hip fracture were considered for inclusion. In order to be included in the study, patients had to be residents in the South West of England. Patients who were unable to walk before hip fracture, and/or were not expected to live until 120 days after their operation (post-operative day 120 (POD 120)) and/or for whom a femoral stem not of a proven stem design (in line with NICE clinical guidance on hip fracture management) would be used, were not eligible to take part in the study.

A total of 244 patients were recruited between November 2019 and April 2022 and randomised in a 1:1 ratio, in theatre, to have their operation performed either using the SPAIRE or the lateral surgical approach. Randomisation was stratified by hospital site and by cognition level (impaired vs non-impaired) from information gathered by the research nurse in the patient’s records. Data collection took place at screening in order to determine eligibility, at baseline (pre-surgery), at 3 days after surgery (POD 3) and at 120 days after surgery (POD 120). Full details of the trial background and rationale, design and sample size calculation have been previously reported [3].

### Intervention

In the SPAIRE approach, the tendon insertions of piriformis, gemellus superior, obturator internus and gemellus inferior muscles are spared, and the extensive abductor muscle insertions of gluteus medius and gluteus minimus onto the greater trochanter are left undisturbed. Full details of the technique are published elsewhere [6]. Having completed a scoping review [7] comparing the use of the traditional posterior approach versus other approaches, funding was secured from the National Institute for Health and Care Research (NIHR) Research for Patient Benefit (RfPB) programme (PB-PG-0817–20,039) to conduct a definitive trial comparing the use of the SPAIRE technique against the standard lateral approach (the HemiSPAIRE trial).

### Trial objectives

The primary objective of this randomised controlled trial is to test whether the SPAIRE technique improves post-operative function and mobility, in terms of the Oxford Hip Score (OHS) [8, 9], at 120 days after surgery in adults aged 60 years or older, with a displaced intracapsular hip fracture requiring hemiarthroplasty, compared to the standard lateral approach. Secondary objectives are to (1) test whether the SPAIRE technique results in improved early function, mobility and pain, pain and quality of life at 120 days, length of hospital stay and complication rates and mortality up to 120 days following surgery compared to the standard lateral approach, through collecting secondary outcome measures; (2) investigate how patients experience the recovery period after surgery, and investigate mechanisms of recovery, including experience of post-operative pain and engagement in physiotherapy, which may contribute to any differences between trial arms, by conducting a qualitative study with a sub-sample of patients in each trial arm; and (3) work with patients and carers with relevant lived experience to ensure the conduct and outputs of the study are relevant and useful to patients who receive hemiarthroplasty surgery. This article focuses on the analyses planned to address the primary objective and secondary objective (1).

### Flow of patients

**Fig. 1:**
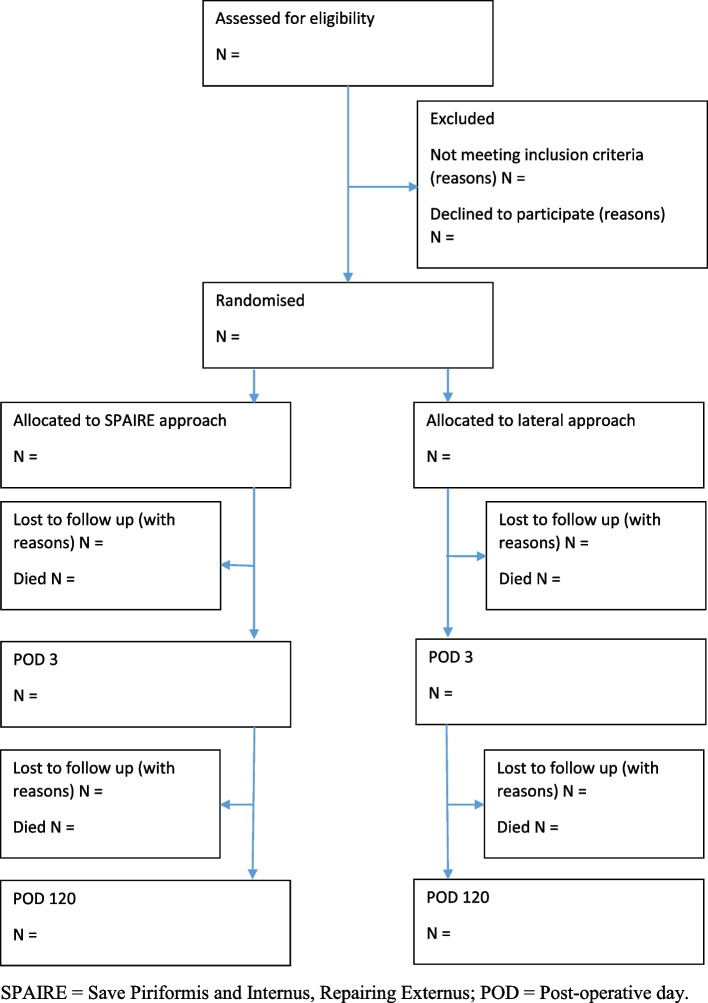
Flow of participants through the HemiSPAIRE trial

The flow of participants through the trial will be reported in accordance with the CONSORT statement for randomised controlled trials (Fig. 1) [5]. The flow diagram will include the number of eligible and recruited patients, and, by allocated group, the number of patients who continued through the trial, the number withdrawing at each time point, the number lost to follow-up at each time point and the numbers included in the analysis.

### Withdrawal/follow-up

Every effort is made to minimise withdrawal and loss to follow-up. Pre-fracture characteristics will be compared between participants who do and do not provide follow-up data on the primary outcome at POD 120, by trial arm and overall. Characteristics will be summarised separately for participants who survive and provide follow-up data; participants who survive and do not provide follow-up data, and participants who do not survive to POD 120. Reasons for withdrawal or loss to follow-up are documented wherever possible.

### Integrity of data

The trial manager is carrying out central monitoring of the data for any errors and omissions to maintain data integrity, as per the trial data management plan. A 10% check of all participant data is also being performed. Data queries are raised directly with hospital sites by the trial manager for discussion and reconciliation. A second, blinded, quality check will be undertaken prior to database lock by the trial statistician. An audit log of these checks is stored in the Trial Master File. Range and sense checks will be performed on all variables prior to commencing statistical analyses.

## Outcomes

### Primary outcome

The primary outcome is the Oxford Hip Score (OHS) at POD 120. The trial is powered to detect a difference between allocated trial arms in the OHS of 5 points [10], with 90% power and a two-sided type 1 error rate of 0.05, assuming the standard deviation (SD) of the OHS is 10. The total loss to follow-up was originally estimated to be 25%, giving a total recruitment target of 228 participants. Following monitoring of follow-up rates by the trial team during the trial, this rate was revised to 30%, resulting in an updated recruitment target of 244 participants. The OHS gives a total score between 0 and 48 [8, 9] and, due to COVID-19 restrictions at the time of this study, is collected over the phone by research nurses. The OHS can be completed by the participant themselves or by proxy from a family member or carer if the participant is unable to answer the questions.

### Secondary outcomes

- Level of function and mobility measured at POD 3 (and POD 120 if possible), using the De Morton Mobility Index (DEMMI) test [11].
- Early mobility, measured using the Cumulated Ambulation Score (CAS) at POD 3 [12].
- Level of pain, using a numeric pain rating scale (NPRS) at POD 3 and POD 120.
- Health-related quality of life (EuroQol EQ-5D-5L) at POD 120 [13].
- Acute and total length of hospital stay.
- Specific hip-related complications: dislocation, peri-prosthetic fracture, infection within 120 days of the operation and need for re-operation.
- Discharge destination.
- Place of residence at POD 120.
- Mortality within 120 days of operation.

## General analyses principles

### Participant population

Comparisons of the outcomes between the trial arms will use the all-randomised population, under the intention-to-treat (ITT) principle with all participants analysed according to the trial arm they were randomised to. Adherence will be assessed based on the percentage of operations that were performed using the approach that was allocated at randomisation, and percentage of follow-up assessments that were completed within the pre-specified data collection windows. All deviations from the study protocol will be reported. The number of participants with protocol deviations will be reported descriptively by trial arm. The pre-specified data collection window for the early post-operative follow-up is POD 3 or POD 4. For the POD 120 follow-up, the data collection window is between POD 110 and 130.

Outcome measurements collected outside the pre-specified windows will be excluded from the main analyses. Additional analyses will be carried out including these measurements. Main analyses of all primary and secondary outcomes at POD 3 and POD 120 will be on the complete case data and will be repeated to include imputed data, using multiple imputation.

Where an outcome can be completed by proxy (i.e. OHS and EQ-5D-5L), these proxy data will be included together with participant-reported data in the main analyses. Those participants for whom outcome data collection was by proxy will be excluded in an additional analysis.

### Levels of confidence and *p*-values

All statistical tests and confidence intervals (CIs) will be two-sided. All between-group comparisons will be presented as the estimate with two-sided 95% CI and *p*-value. Statistical significance will be set at the 5% level. Results of all between-group comparisons of continuous outcomes will be checked for validity using bootstrap methods.

### Unadjusted and adjusted analyses

Unless stated otherwise, analyses of all outcomes will be adjusted for the stratification variables hospital site and cognition level, and pre-fracture characteristics age (continuous), gender, place of residence (categorical) and co-morbidities (American Society of Anesthesiologists (ASA) score, grouped into 1 or 2, 3, 4 +). Unadjusted analyses will also be reported. The adjusted analyses will be considered to be the main analyses.

### Multiple testing

No adjustments will be made for multiple testing, and the secondary outcomes will be considered exploratory.

### Missing data

For the primary outcome and secondary outcomes, at each follow-up time point, the percentage of missing data will be reported, by trial arm and overall.

Every effort will be made to collect outcome data within the pre-specified data collection windows. However, data collection will still be attempted outside these windows and the data will be used for the purposes of additional analyses.

Where participants have missing data on a subset of items for a given measure a decision will be made on the minimum number of items that should be responded to for a total score to be obtained. This will be done in advance of the database being locked and blind to the knowledge of which arm the participants are allocated to. Any measure-specific rules for obtaining total scores when items are missing will be used. Specifically, for the primary outcome OHS, if one or two items are missing these will be replaced with the mean of the remaining items, and a total score calculated. If more than two items are missing, a total score will not be calculated and that participant will not be included in the main analysis [14].

The main analyses will be based on complete case data. Additional analyses will be carried out for all outcomes, based on multiple imputation. Multiple imputation will be used to impute missing data on outcomes, under the assumption that data are missing at random according to Rubin’s rules, i.e. that missingness is accounted for by other variables within the dataset [15]. Missing data will be imputed using the chained equation approach. Predictive mean matching, in which imputed values are sampled only from the observed values, will be used [16]. A total of 50 imputed datasets will be generated. Variables used to impute missing data will include all outcomes at all follow-up time points, trial arm status, stratification variables, and variables included as adjustment factors in the regression models fitted to outcomes. While all participants will be included in the imputation process, no outcomes will be imputed for participants who die before the outcome could have been assessed. That is, if a participant dies before POD 3, no outcomes will be imputed for that participant; if a participant dies between POD 3 and POD 120, POD 3 outcomes will be imputed if missing; if a participant survives beyond the POD 120 follow-up all missing outcomes will be imputed. Note: this principle is used in the process of obtaining multiple imputed datasets but does not apply to the *composite* approach used in additional analyses, described later in this article, in which those participants who have died before the outcome can be assessed are given a ‘worst case’ score for that outcome.

The multiple imputation will be carried out using the *mi* suite of commands in Stata. All statistical analyses will be carried out using Stata version 17.0 or higher [17].

### Presentation of comparative analyses

For continuous outcomes (including the primary outcome), results will be presented as means and SDs in the two trial arms, crude (unadjusted) mean differences, and adjusted mean differences with 95% CIs and *p*-values. In addition, the effect size (adjusted mean difference divided by pooled SD) will be reported for the OHS at POD 120, for the main analysis only. For time-to-event outcomes, frequencies of events in the two arms will be presented, crude hazard ratios (HRs), and adjusted HRs with 95% CIs and *p*-values.

For binary outcomes, numbers and percentages in the two arms will be presented, with crude odds ratios (ORs) and adjusted ORs with 95% CIs and *p*-values.

## Proposed analyses

### Baseline

Baseline participant characteristics summarised by trial arm status and overall will include: gender, age, ethnicity, cognition level, place of residence, co-morbidities (ASA score), pre-injury level of function and pain from OHS, pre-injury quality of life from EQ-5D-5L and hospital site. Means and SDs (or medians and interquartile ranges) will be presented for continuous variables and numbers and percentages for categorical variables. No formal comparisons of baseline characteristics will be made between trial arms, as recommended by the CONSORT 2010 guidelines for reporting parallel group randomised controlled trials [5].

### Planned main analysis of the primary outcome

The primary outcome (OHS) was originally going to be analysed using linear regression. Review of trial data by the Trial Steering Committee identified differential survival between trial arms. Consequently, it was agreed that planned analysis of the primary outcome (OHS) will use a survivor average causal effect (SACE) approach, which allows estimation of the effect of surgical approach on outcomes in the population of people who would have survived regardless of what surgical approach they received. The method of principal stratification [18] will be used, which assumes that the survival status for each participant allocated to SPAIRE, D(S) or to lateral, D(L), are conditionally independent given a set of baseline covariates (X), which are predictive of survival. It also assumes that conditional on a participant surviving when randomised to SPAIRE, and the covariates, their survival status under lateral is independent of the outcome Y(SPAIRE) and vice versa (swapping SPAIRE and lateral). The effect of surgical approach can then be estimated by following the steps outlined below:

- Fit a logistic regression model in those randomised to SPAIRE where survival = 1 and death = 0, with X as covariates. Use this model to calculate a fitted probability of survival for *all* participants, denoted P_S.
- Fit a logistic regression model in those randomised to lateral where survival = 1 and death = 0, with X as covariates. Use this model to calculate a fitted probability of survival for *all* participants, denoted P_L.
- Fit a weighted linear (for continuous outcomes) or logistic (for binary outcomes) regression model (using weights P_L for SPAIRE group and P_S for lateral group) to estimate the effect of SPAIRE compared to lateral on the outcome Y in the principal strata who would survive under allocation to SPAIRE or lateral.

Steps a and b are performed once, to obtain the weights. These weights are then used in step c), which is performed for each outcome.

The set of baseline covariates X that will be used in steps a and b are gender, age, cognitive impairment, place of residence and co-morbidities (ASA score), all known to be predictive of survival in this patient group and currently used in the Nottingham Hip Fracture Score model to provide surgeons with the ability to calculate a mortality risk online for their patients [19]. The remaining baseline variable that is collected in the trial is ethnicity, and this will be explored as a possible predictor for inclusion.

In step c, the OHS at POD 120 will be compared between trial arms using weighted linear regression, adjusting for the stratification variables, and the pre-fracture characteristics: age, gender, place of residence and ASA score.

### Planned analyses of the secondary outcomes

Secondary outcomes were originally going to be analysed using linear (continuous outcomes), or logistic (binary outcomes) regression. As described above for the OHS, following the identification of differential survival between trial arms, secondary outcomes (excluding surgical complications and mortality) will be analysed using the SACE method, as described above. In step c of the approach, continuous secondary outcomes will be analysed in the same way as the OHS. The binary secondary outcomes of discharge destination (i.e. whether the same as pre-fracture place of residence) and place of residence at 120 days (i.e. whether the same as pre-fracture place of residence) will be compared between trial arms using weighted logistic regression. For each of these outcomes, the number of events will be checked when considering adjustment factors to be included in the analyses, additional to the trial arm.

Frequencies of surgical complications within 120 days follow-up will be presented by type, in the two trial arms (i.e. the number of participants in each arm who have each type of complication, the three most common expected complications being: dislocations, peri-prosthetic fractures and infections). Kaplan Meier plots will be used to show these events (either complications of all types, or separated by type, depending on the numbers of complications occurring) in the two trial arms and to visually check the assumption of proportional hazards. If there is no clear evidence of non-proportional hazards, and sufficient numbers of complications in the two arms to warrant formal comparison, Cox regression will be used to analyse time to the first complication. Censored participants will be those who are lost to follow-up, who drop out, die, or reach 120 days before having a complication.

Frequencies of deaths prior to POD 3 follow-up and within 120 days after surgery will be reported in the two trial arms.

Kaplan Meier plots will be used to show deaths within 120 days follow-up, in the two trial arms and to visually check the assumption of proportional hazards. If there is no clear evidence of non-proportional hazards, Cox regression will be used to analyse time to death.

The number of events will be checked before considering adjustment factors to be included in the Cox regression models, additional to the trial arm.

### Planned additional analyses of the primary and secondary outcomes

The following additional analyses will be conducted.

#### Analyses of trial data (not using multiple imputed datasets)

The primary analysis is a SACE analysis of the complete case data, based on those participants who would have survived regardless of which surgical approach they received.

The following additional analyses will be carried out on the trial data:

1. Analysis of the primary outcome and each of the secondary outcomes (excluding surgical complications and mortality), using linear (for continuous outcomes) and logistic (for binary outcomes) regression, setting those participants who have died before the outcome can be assessed to the worst possible score (for continuous outcomes OHS, DEMMI, CAS, NPRS), the score equivalent to being dead (for EQ-5D-5L), the worst score observed among all participants (for lengths of stay), or the worst category (for binary outcomes discharged to pre-fracture residence, and living at pre-fracture place of residence at POD 120)—a *composite* approach. The population of interest is comprised of all participants regardless of whether they survived or not. Table 1 gives details of how this approach will be applied for each outcome.
2. Analysis of the primary outcome and each of the secondary outcomes, using linear and logistic regression, among those participants who survived—a *survivors* analysis. The population of interest is comprised of participants who survived under the surgical approach they received.
3. Analysis of the primary outcome and each of the secondary outcomes, including any outcomes collected outside the pre-specified data collection windows, using a SACE analysis.
4. Analyses of all outcomes that can be completed by proxy, excluding those participants for whom outcome data collection was by proxy, using a SACE analysis.

**Table 1:** Details of how the composite approach will be applied for each outcome

Outcome	Scoring range/categorisation of outcome	Score/category that participants who die before the outcome can be assessed are allocated to
OHS	0 (worst) to 48 (best)	0
DEMMI	0 (worst) to 100 (best)	0
CAS	0 (worst) to 18 (best)	0
NPRS	0 (no pain) to 10 (worst possible pain)	10
EQ-5D-5L	Less than 0 to 1 (0 being value of a health state equivalent to dead)	0
Length of stay (acute)	Number of days of acute stay	The maximum number of days observed among all participants
Length of stay (total)	Number of days of total stay	The maximum number of days observed among all participants
Discharged to same place of residence as pre-fracture	Participant’s discharge destination and pre-fracture place of residence reported and participant classified as either being discharged to their pre-fracture place of residence (1) or to a different place (0)	0
Living in same place of residence at 120 days as pre-fracture	Participant’s place of residence at 120 days and pre-fracture place of residence reported and participant classified as either living at their pre-fracture place of residence (1) at 120 days or elsewhere (0)	0

*OHS* Oxford Hip Score, *DEMMI* De Morton Mobility Index, *NPRS* Numeric Pain Rating Scale, *EQ5D-5L* five-level EQ-5D, *CAS* Cumulated Ambulation Score

#### Multiple imputation analyses

The following additional analyses will be carried out based on multiple imputed datasets:

5. Analysis of the primary outcome and each of the secondary outcomes using a SACE analysis, based on those participants who would have survived regardless of which surgical approach they received.
6. Analysis of the primary outcome and each of the secondary outcomes, using linear and logistic regression, setting those participants who have died before the outcome can be assessed to the worst possible score (for continuous outcomes) or category (for categorical outcomes) for that outcome-a *composite* approach. The population of interest is comprised of all participants regardless of whether they survived or not (see Table 1 for details of how this approach will be applied for each outcome).
7. Analysis of the primary outcome and each of the secondary outcomes, using linear and logistic regression, among those participants who survived-a *survivors* analysis. The population of interest is comprised of participants who survived under the surgical approach they received.

### Adverse events

Any potential adverse events (AEs) will be recorded and managed in accordance with the study adverse event protocol. The number of AEs occurring in each trial arm will be reported, by type. The number and percentage of participants having one or more AE, and the number and percentage having one or more serious adverse event (SAE), will also be reported.

## Discussion

The article reporting the protocol for this randomised controlled trial included a brief outline of the planned statistical analyses, which were subsequently further developed and modified after the start of the trial. This detailed statistical analysis plan was written during the delivery period of the HemiSPAIRE trial and was finalised prior to final data collection.

By publishing our detailed statistical analysis plan for a randomised controlled trial of a surgical intervention in an older population, we hope that it may be of use to other teams developing plans for similar trials, with similar considerations to be made, particularly regarding comparing outcomes between groups where there might be differential survival or a significant risk that participants do not survive to, or are not able to be assessed at, the primary follow-up time point.

## Data Availability

Not applicable.

## Abbreviations

AE: Adverse event
ASA: American Society of Anesthesiologists
CAS: Cumulated Ambulation Score
CI: Confidence interval
CONSORT: Consolidated Standards of Reporting Trials
DEMMI: De Morton Mobility Index
EQ-5D-5L: EuroQol 5-level EQ-5D
HR: Hazard ratio
ICH: International Conference on Harmonisation
NICE: National Institute for Health and Care Excellence
NIHR: National Institute for Health and Care Research
NPRS: Numeric pain rating scale
OHS: Oxford Hip Score
OR: Odds ratio
POD: Post-operative day
RfPB: Research for Patient Benefit
SACE: Survivor average causal effect
SAE: Serious adverse event
SD: Standard deviation
SPAIRE: Save Piriformis and Internus, Repairing Externus
